# Regulatory Lymphocytes Are Key Factors in MHC-Independent Resistance to EAE

**DOI:** 10.1155/2014/156380

**Published:** 2014-04-27

**Authors:** Nieves Marín, Miriam Mecha, Carmen Espejo, Leyre Mestre, Herena Eixarch, Xavier Montalban, José C. Álvarez-Cermeño, Carmen Guaza, Luisa M. Villar

**Affiliations:** ^1^Multiple Sclerosis Unit, Immunology and Neurology Departments, Hospital Universitario Ramón y Cajal, IRYCIS, Carretera de Colmenar Km 9.100, 28034 Madrid, Spain; ^2^Red Española de Esclerosis Múltiple (REEM), RETICS, Fondo de Investigación Sanitaria, Instituto de Salud Carlos III, Ministerio de Economía y Competitividad, Madrid, Spain; ^3^Neuroimmunology Group, Department of Functional and System Neurobiology, Instituto Cajal, Consejo Superior de Investigaciones Científicas, Avenida Doctor Arce 37, 28002 Madrid, Spain; ^4^Servei de Neurologia-Neuroimmunologia, Centre d'Esclerosi Múltiple de Catalunya, Vall d'Hebron Institut de Recerca, Hospital Universitari Vall d'Hebron, Passeig de la Vall d'Hebron 119-129, 08035 Barcelona, Spain; ^5^Universitat Autònoma de Barcelona, Bellaterra, 08193 Cerdanyola del Vallès, Spain; ^6^Department of Medicine, Universidad de Alcalá, Plaza de San Diego s/n, Alcalá de Henares, 28801 Madrid, Spain

## Abstract

*Background and Objectives*. Resistant and susceptible mouse strains to experimental autoimmune encephalomyelitis (EAE), an inducible demyelinating experimental disease serving as animal model for multiple sclerosis, have been described. We aimed to explore MHC-independent mechanisms inducing resistance to EAE. *Methods*. For EAE induction, female C57BL/6 (susceptible strain) and CD1 (resistant outbred strain showing heterogeneous MHC antigens) mice were immunized with the 35–55 peptide of myelin oligodendrocyte glycoprotein (MOG_35−55_). We studied T cell proliferation, regulatory and effector cell subpopulations, intracellular and serum cytokine patterns, and titers of anti-MOG serum antibodies. *Results*. Upon immunization with MOG_35−55_, T lymphocytes from susceptible mice but not that of resistant strain were capable of proliferating when stimulated with MOG_35−55_. Accordingly, resistant mice experienced a rise in regulatory B cells (*P* = 0.001) and, to a lower extent, in regulatory T cells (*P* = 0.02) compared with C57BL/6 susceptible mice. As a consequence, MOG_35−55_-immunized C57BL/6 mice showed higher percentages of CD4+ T cells producing both IFN-gamma (*P* = 0.02) and IL-17 (*P* = 0.009) and higher serum levels of IL-17 (*P* = 0.04) than resistant mice. *Conclusions*. Expansion of regulatory B and T cells contributes to the induction of resistance to EAE by an MHC-independent mechanism.

## 1. Introduction

Multiple sclerosis (MS) is a chronic inflammatory disease of the central nervous system (CNS). Numerous evidences strongly suggest that it is an autoimmune disease in which activated T cells enter the CNS and trigger an inflammatory cascade resulting in demyelination and axonal loss. Different genetic and environmental factors have shown to play a role in MS susceptibility, being class II alleles of the major histocompatibility complex (MHC), the more closely associated genes [1], and infectious agents such as Epstein-Barr virus, the environmental factors that have been more clearly associated with MS susceptibility [2]. Conversely, high serum levels of vitamin D have a protective role, delaying the appearance of a second demyelinating event after a clinically isolated syndrome [3]. It has been described that this action can be mediated by upregulation of regulatory T cells (Treg) [4] and B cells (Breg) [5]. It was also described that MS patients suffer from a peripheral B cell tolerance defect that may be attributable to impaired Treg function [6] and that proliferation of Breg induced by intestinal helminth infections ameliorates MS course [7].

The role of regulatory cells in demyelinating diseases has been also explored in experimental autoimmune encephalomyelitis (EAE), a widely used animal model of MS in which T cell mediates CNS demyelination [8]. EAE can be induced in several species by immunization with myelin antigens or via adoptive transfer of myelin-reactive T cells [9, 10]. Adoptive transfer of Breg reduced significantly the severity of EAE by inhibiting Th1 and Th17 inflammatory responses [11]. Likewise, expansion of Treg diminished the infiltration of inflammatory cells into the CNS and improved clinical signs of EAE [12]. These data strongly suggest that deficits in regulatory pathways may contribute to the induction of autoimmune reactivity in EAE. These experimental models may be useful tools to identify immunological mechanisms mediating a protective role in demyelinating diseases.

Several strains of mice are resistant to develop EAE as mice are asymptomatic following immunization with myelin antigens [13]. It was argued that a poor recognition of encephalitogenic peptides by MHC molecules might contribute to resistance to EAE. However, other immune mechanisms may also contribute to inducing resistance to the disease. We explored them in two mouse strains, CD1 and C57BL/6, that are, respectively, resistant and susceptible to EAE induced by the myelin oligodendrocyte glycoprotein 35–55 peptide (MOG_35–55_). Since CD1 is an outbred strain, these mice do not share a unique MHC haplotype, which makes this model useful to study MHC-independent mechanisms that mediate resistance to this experimental model of MS. We explored Breg and Treg in both strains and studied if variations in regulatory cells associated with downregulation of effector immune mechanisms.

## 2. Materials and Methods

### 2.1. Mice

Eight-to-ten-week-old female C57BL/6J mice, which are susceptible to EAE induction by MOG_35–55_ immunization, were purchased from Harlan (Barcelona, Spain). Age- and sex-matched CD1 mice that are resistant to EAE induction were obtained from our own conventional barrier protection breeding colony (Cajal Institute, CSIC, Madrid, Spain). Mice were housed under standardized light- and climate-controlled conditions and were fed with standard chow and water ad libitum. Experiments were done in compliance with the guidelines of animal care set by the European Union (86/609/EEC), and all animal protocols were approved by the Cajal Institute animal welfare committee (Protocol N IORG0006540).

### 2.2. EAE Induction

For immunization C57BL/6 and CD1 mice were injected subcutaneously on day 0 with 200 *μ*L of an emulsion containing 300 *μ*g of MOG_35–55_ (CNB, CSIC, Madrid, Spain) and 800 *μ*g of heat killed* Mycobacterium tuberculosis* H37RA (Difco, BD Diagnostics, MD) emulsified in incomplete Freund adjuvant oil (Sigma-Aldrich, St. Louis, MO). In addition, the mice received 100 ng of* Bordetella pertussis* toxin (Sigma-Aldrich) intraperitoneally on days 0 and 2 after immunization (p.i.). Mice from both strains C57BL/6 or CD1 were immunized in the same way using the 139–151 peptide of proteolipid protein (PLP_139–151_) (Proteomics Section, Universitat Pompeu Fabra, Barcelona, Spain) for comparison with MOG_35–55_ as C57BL/6 mice do not develop EAE following immunization with PLP peptide. Age-matched naïve C57BL/6 or CD1 mice were considered as control groups.

Mice were monitored daily, and clinical signs of EAE were graded as follows: Grade 0, normal; Grade 1, flaccid tail; Grade 2, mild hind-limb weakness; Grade 3, severe hind-limb weakness; Grade 4, hind-limb paralysis; Grade 5, hind-limb paralysis and partial fore-limb weakness.

### 2.3. Experimental Procedure

On the basis of previous reports [14] we selected day 21 p.i. as the time point in which experimental procedures were performed. Mice were anaesthetized by intraperitoneal administration of EuTalender (Normonlab, Madrid, Spain). Blood samples were collected by intracardiac puncture. Serum was obtained and frozen at −80°C until soluble cytokines and antibodies were assayed. Spleens were removed and splenocyte suspensions were generated by grinding spleens through a wire mesh. Lymphocyte subsets and intracellular cytokine production were tested in freshly isolated splenocytes.

### 2.4. Splenocyte Proliferation Assay

The splenocytes were seeded in 96-well plates at a cell density of 2 × 10^5^ cells/well in Iscove's modified Dulbecco's medium (IMDM; PAA Laboratories GmbH, Pasching, Austria) supplemented with 10% HyClone FetalClone I (Thermo Fisher Scientific, Waltham, MA, USA), 50 *μ*mol/L of 2-mercaptoethanol (Sigma Chemical), 2 mmol/L of glutamine, 50 U/mL of penicillin, and 50 mg/mL of streptomycin; the last three chemicals were obtained from Gibco BRL (Paisley, UK). For splenocyte activation, we used 5 *μ*g/mL of MOG_35–55_, PLP_139–151_, or phytohaemagglutinin (PHA; Sigma Chemical). Cells were incubated in a humidified atmosphere at 5% CO_2_ and 37°C for 3 days, the last 18–20 h in the presence of 1 *μ*Ci/well of [^3^H]-thymidine (PerkinElmer Inc., USA). The levels of incorporated radioactivity were determined using a beta-scintillation counter (Wallac, Turku, Finland). The stimulation index (SI) was expressed as the mean of the counts per minute (cpm) of five replicates from each mouse and culture condition divided by the mean cpm of the baseline control replicates. The results are expressed as the mean value ± standard error of the mean (SEM) of the SI per group of mice.

### 2.5. Study of B and T Cell Subsets

We performed this study in series of seven mice per condition. To study lymphocyte subsets, cells were resuspended at 10^6^/mL and incubated with rat anti-mouse CD16/32 Fc receptor (FcR) (CD16/CD32) during 10 minutes to avoid unspecific staining. Then, they were incubated with the appropriate combinations of monoclonal antibodies (BD Pharmingen, San Diego, CA) for 20 minutes at 4°C. Cells were washed with PBS and flow-cytometry analysis was performed on FACScanto II (Becton Dickinson) and analyzed using the DIVA software (Becton Dickinson). As strategy for flow-cytometry analysis, an initial region was set around cells expressing intermediate to high CD45 with low-to-intermediate side scatter (P1) and then a second region was set on the forward/side scatter dot plot to exclude debris or apoptotic cells (P2). Only cells included in P1 and P2 regions were accepted for analysis. We used the following monoclonal antibodies: anti-CD4-FITC, anti-CD8a-PE, anti-CD45-Cy5.5PerCP, anti-CD25-APC, anti-B220-FITC, anti-CD1d-PE, and anti-CD5-APC, and isotype controls conjugated with FITC, PE, Cy5.5PerCP, and APC.

### 2.6. Analysis of Intracellular Cytokine Production

Splenocytes were resuspended (10^6^ cells/mL) in complete medium [RPMI supplemented with 2 mM glutamine, 2 mg/mL gentamycin (all from Gibco BRL (Paisley, UK)), and 10% fetal calf serum (Biowhittaker technologies, MD, USA)] and the cells were cultured for four hours with PMA (50 ng/mL; Sigma-Aldrich, St. Louis, MO), ionomycin (500 ng/mL; Sigma-Aldrich), and monensin (2 *μ*M; eBioscience, San Diego, CA).

For IL-17A and IFN-gamma detection, FcR were blocked with anti-CD16/CD32 antibody (BD Pharmingen) for 10 minutes at room temperature before cell surface staining. Cells were stained with antibodies against surface antigens for 30 min at 4°C using predetermined optimal concentrations of each antibody. Stained cells were fixed and permeabilized using a Cytofix/Cytoperm kit (BD Pharmingen), according to the manufacturer's instructions, and stained with anti-IL-17A-PE and anti-IFN-gamma-APC for 30 min at 4°C. After washing cells, flow-cytometry analysis was performed on FACScanto II (Becton Dickinson) and analyzed using the DIVA software (Becton Dickinson).

### 2.7. Quantification of Serum Cytokines

Serum cytokines were determined by a Milliplex map mouse cytokine panel (Millipore, Billerica, MA) following manufacturer instructions. Data were analyzed in a TM100 cytometer (Labscan, Gliwice, Poland).

### 2.8. Detection of Anti-MOG_35–55_ Antibodies

Maxisorp microtiter plates (Nunc, Roskilde, Denmark) were coated overnight at 4°C with 100 *μ*L/well of mouse MOG_35–55_ peptides (CNB, CSIC) at a concentration of 10 *μ*g/mL in 0.1 M carbonate buffer, pH 9.7. Plates were washed with PBS containing 0.02% Tween 20 and blocked with 0.1 M carbonate buffer, pH 9.7, containing 5% BSA for 1 h at 37°C. Then, plates were incubated with triplicate serum samples diluted at 1/200 for 1 h at 37°C and washed and incubated with biotinylated anti-mouse IgM (Sigma-Aldrich) or anti-mouse IgG (Jackson Immunoresearch, Suffolk, UK) for 1 h at 37°C. After this, they were washed and incubated with streptavidin-horseradish peroxidase (Roche, Basel, Switzerland) for 30 min at 37°C. After a final wash, the reaction products were visualized using OPD (Sigma-Aldrich) as a substrate and read at 492 nm with a microplate reader (Thermo Fisher, Cambridge, UK).

### 2.9. Statistical Analysis

Results were analyzed with Prism 5.0 statistical package. We used Mann-Whitney *U* test for comparisons between groups. *P* values lower than 0.05 were considered significant.

Experiments were always performed in two independent series of mice using four animals per condition. We combined them for the final statistical analysis. Results are always expressed as mean ± SEM.

## 3. Results 

We explored regulatory and effector immune mechanisms in CD1 and C57BL/6 mice immunized with MOG_35–55_, using as controls nonimmunized mice and mice immunized with PLP_139–151_, a myelin peptide that is not capable of inducing EAE in any of those mouse strains.

### 3.1. Splenocyte Proliferative Response

**Figure 1:**
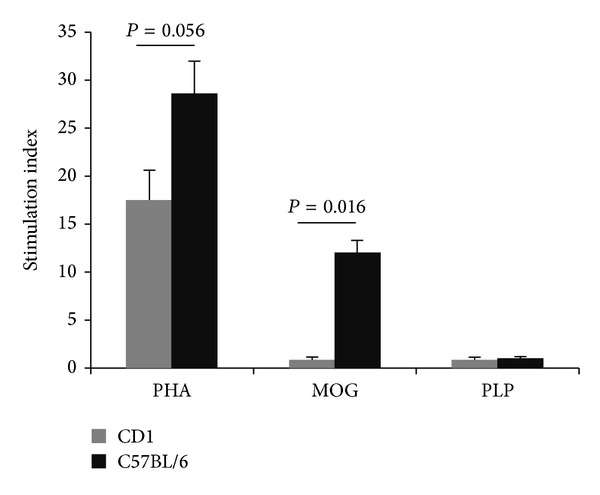
Proliferative capacity of splenocytes from C57BL/6 (susceptible strain) and CD1 (resistant strain) mice. We analyzed proliferative capacity in series of seven mice per condition. Splenocytes from resistant and susceptible mice were stimulated with a polyclonal stimulus (PHA), MOG_35–55_, or PLP_139–151_. Susceptible mice showed a higher proliferative capacity upon PHA stimulation and a statistically significant augmented proliferation upon MOG stimulation. Conversely, splenocytes from both strains did not proliferate when the stimulus used was 139–151 PLP peptide.

We tested the polyclonal and antigen-specific proliferative capacity of splenocytes 21 days after the immunization. A diminished proliferation showing a clear statistical trend (*P* = 0.056) was detected in the resistant strain when a polyclonal stimulus such as PHA was used (Figure 1). Moreover, upon antigen-specific stimulation, the splenocytes from CD1 resistant mice did not proliferate, while those of C57BL/6 susceptible mice did (*P* = 0.016, Figure 1). Control study using irrelevant PLP_139–151_ peptide as stimulus did not render proliferation in any of the strains. All these data indicate that despite their heterogeneous MHC antigens, all CD1 resistant mice are unable to develop a T cell response against the MOG_35–55_ encephalitogenic peptide. We next aimed to explore immunological mechanisms that contribute to this phenomenon.

### 3.2. B Cell Subsets

**Figure 2:**
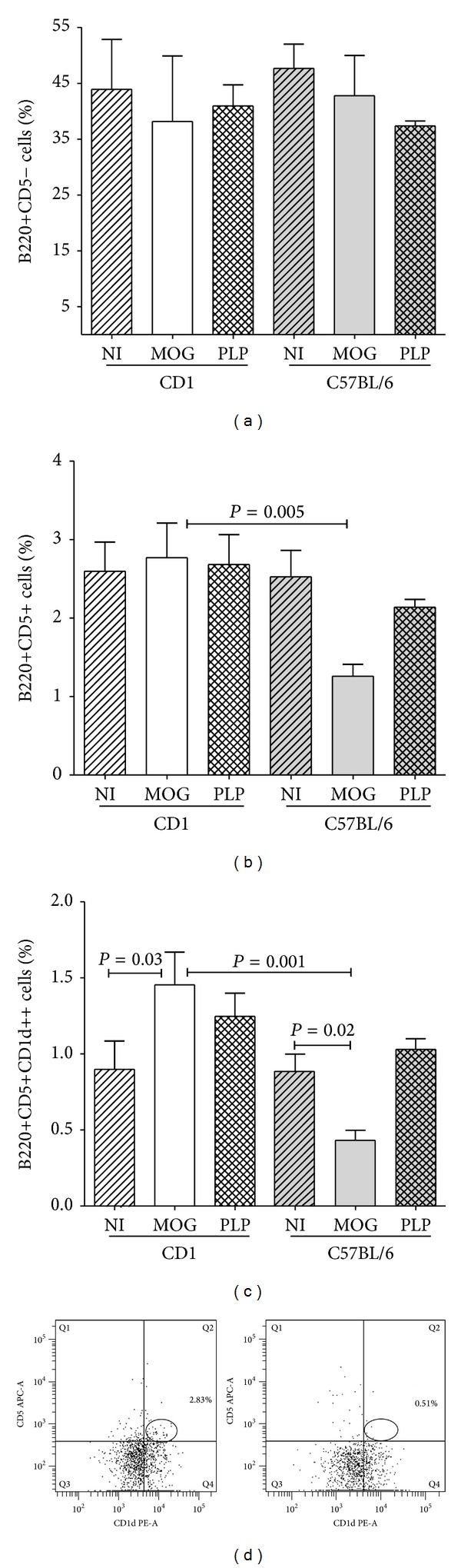
Effector and regulatory B cells in spleens of C57BL/6 and CD1 mice. We analyzed these cells in series of seven mice per condition. (a) B2 cells (B220+CD5−) did not change significantly in mice immunized with MOG_35–55_ (MOG) or PLP_139–151_ (PLP) when compared with nonimmunized (NI) ones. (b) B1 cell (B220+CD5+) percentage diminished in C57BL/6 mice that developed EAE upon immunization with MOG_35–55_. (c) The B220+CD5+CD1d^hi^ regulatory subset (Breg) increased in resistant CD1 mice immunized with MOG. Conversely, it decreased in susceptible C57BL/6 mice developing EAE after immunization with the same peptide. (d) Representative dot plots showing Breg induced in CD1 (left side) and C57BL/6 (right side) mice upon immunization with MOG_35–55 _peptide. Total B cells were gated by selecting B-220 positive cells and then Breg were identified by CD5 and CD1d^hi^ staining.

We first examined B1 and B2 subsets (Figures 2(a) and 2(b)). We did not found differences in the percentages of T cell dependant B2 cells (B220+CD5−) between resistant and susceptible mice (nonimmunized or immunized with MOG or PLP peptides). Conversely, the percentage of innate B220+CD5+ B cells was downmodulated in C57BL/6 mice after EAE induction by MOG_35–55_ (*P* = 0.005) when compared with CD1 resistant mice immunized with the same peptide. These differences were due to the regulatory B220+CD5+CD1d^hi^ B cell subset (Breg). When exploring these B cells, we detected a decrease of Breg in C57BL/6 mice suffering EAE (*P* = 0.02, Figures 2(c) and 2(d)). Conversely, resistant CD1 strain upregulated this subpopulation after immunization with MOG_35–55_ (*P* = 0.03). These differences between the two strains (*P* = 0.001) were only observed upon MOG_35–55_ immunization. By contrast, Breg remained unchanged in either resistant or susceptible mice immunized with the PLP_139–151_ irrelevant peptide. This strongly suggests that Breg are specifically triggered to prevent the autoimmune process and have an important role in inducing resistance to EAE.

### 3.3. Regulatory T Cells

**Figure 3:**
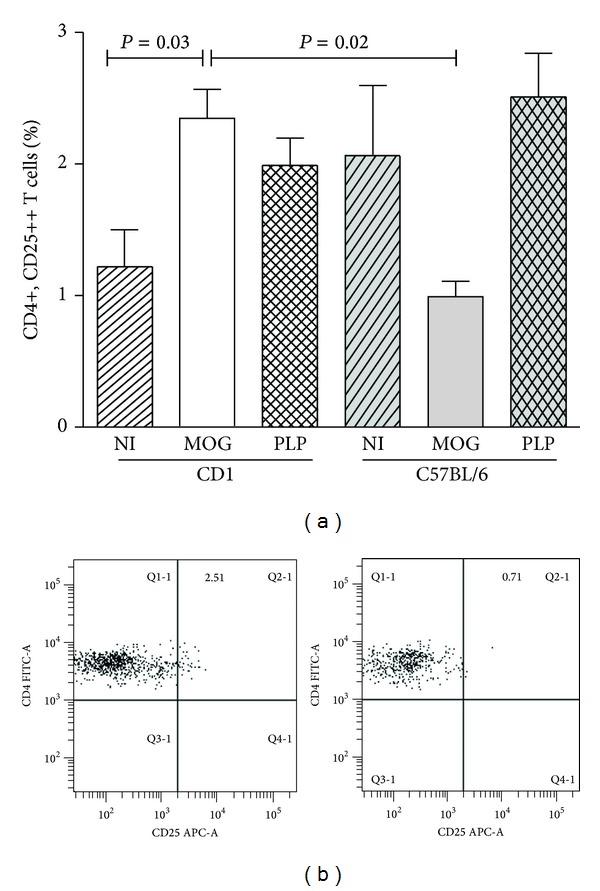
Regulatory T cells in spleens of resistant and susceptible mice. We analyzed these cells in series of seven mice per condition. (a) We studied nonimmunized mice (NI) and mice immunized with MOG_35–55_ (MOG) and with PLP_139–151_ (PLP). CD1 mice upregulated CD4+CD25^hi^ T cells when immunized with MOG or PLP autopeptides. Conversely, Treg percentages did not increase in C57BL/6 mice immunized with MOG that developed EAE. (b) Representative dot plots showing regulatory T cells induced in CD1 (left side) and C57BL/6 (right side) mice upon immunization with MOG_35–55_ peptide.

Breg may contribute to the differentiation of regulatory T cells (Treg) [15]. We also explored this T cell subset (CD4+, CD25^hi^) in our series of mice (Figure 3). Upon immunization with MOG_35–55_ resistant but not susceptible mice upregulated Treg (*P* = 0.028). No differences were found in this T cell subset between resistant and susceptible strains when nonimmunized mice or mice immunized with the PLP_139–151_ peptide were studied. These observations indicate that Treg may also play a role in resistance to EAE shown by CD1 mice immunized with MOG_35–55_.

### 3.4. Effector Responses

**Figure 4:**
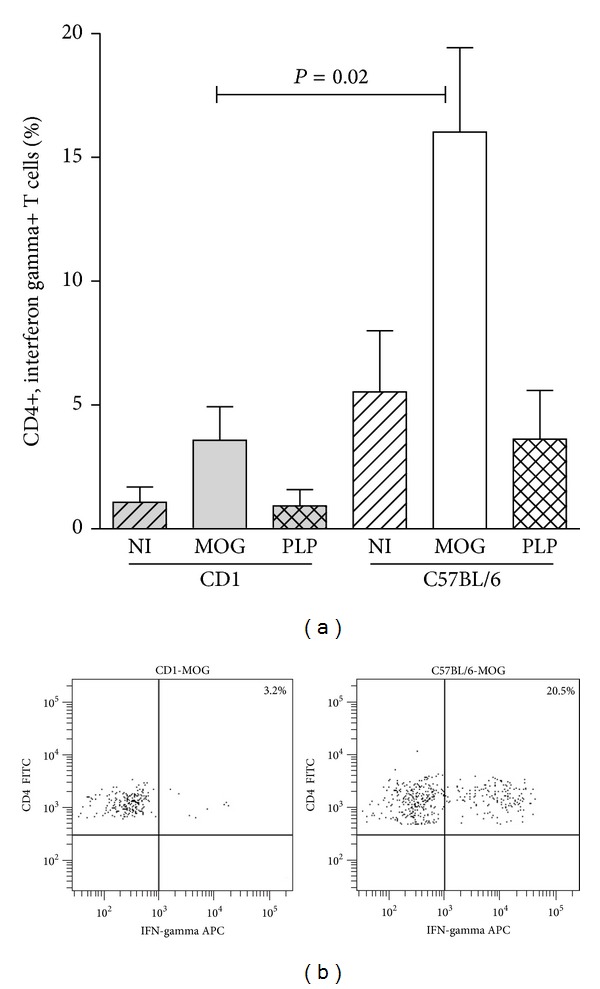
Intracellular production of interferon-gamma (IFN-gamma) by spleen CD4+ T cells. We analyzed these cells in series of seven mice per condition. (a) Splenocytes from C57BL/6 and CD1 mice were cultured in presence of phorbol-12-myristate-13-acetate (PMA) and ionomycin during four hours, and the percentage of CD4+ T cells showing intracellular production of IFN-gamma was analyzed by flow cytometry. We studied nonimmunized mice (NI) and mice immunized with MOG_35–55_ (MOG) and with PLP_139–151_ (PLP). C57BL/6 mice that developed EAE after immunization with MOG_35–55_ experienced a significant increase in the percentage of CD4+ T cells producing IFN-gamma. (b) Representative dot plots of IFN-gamma intracellular staining on CD4+ T splenocytes from CD1 and C57BL/6 mice upon immunization with MOG_35–55_.

**Figure 5:**
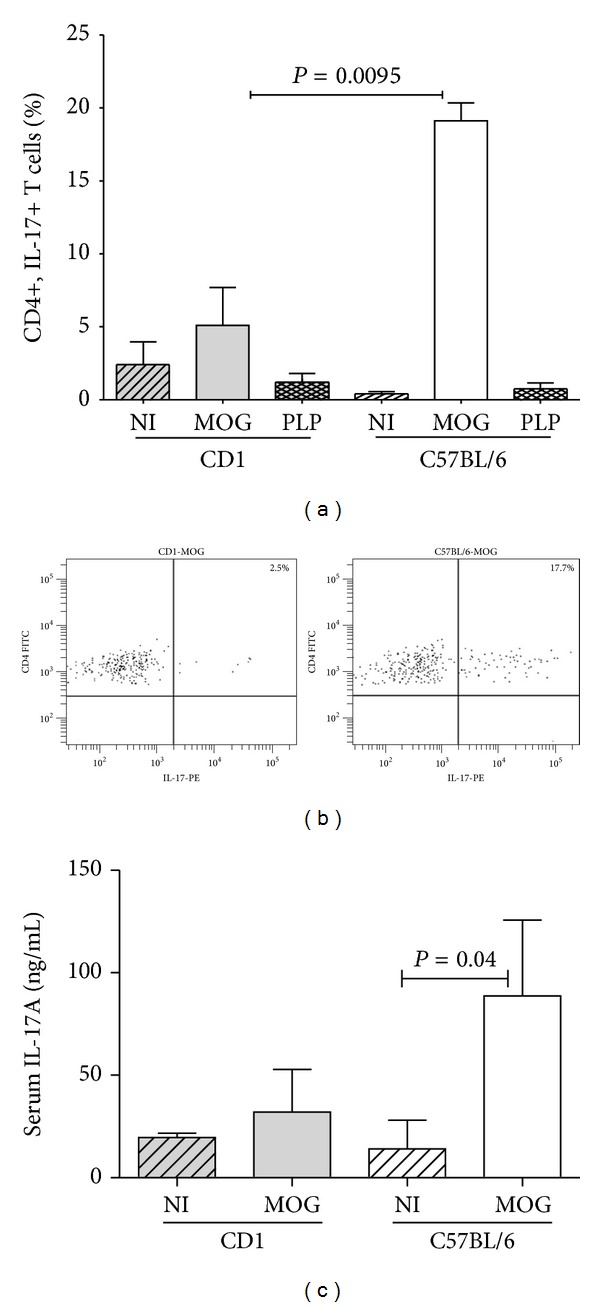
(a) Intracellular production of interleukin-17 (IL-17) by spleen CD4+ T cells. We analyzed these cells in series of seven mice per condition. Splenocytes from C57BL/6 and CD1 mice were cultured in presence of phorbol-12-myristate-13-acetate (PMA) and ionomycin during four hours and the percentage of CD4+ T cells showing intracellular production of IL-17 was analyzed by flow cytometry. We studied nonimmunized mice (NI) and mice immunized with MOG_35–55_ (MOG) and with PLP_139–151_ (PLP). Only C57BL/6 mice that developed EAE after immunization with MOG_35–55_ displayed a significant increase in the percentage of CD4+ T cells producing IL-17. (b) Representative dot plots of IL-17 intracellular production by CD4+ T cells from MOG_35–55_-immunized CD1 and C57BL/6 mice are shown. (c) Serum concentrations of IL-17A in nonimmunized mice (NI) and mice immunized with MOG_35–55_ (MOG).

We did not find differences in the levels of IgM or IgG anti-MOG_35–55_ antibodies or in total CD4+ and CD8+ T cell percentages between the resistant and the susceptible mice (Table 1). To further discriminate different T cell responses, we analyzed Th1 and Th17 subsets. We explored the percentage of CD4+ T cells showing intracellular production of IFN-gamma (Figure 4) and IL-17 (Figure 5) upon stimulation with phorbol-12-myristate-13-acetate (PMA) and ionomycin. Only susceptible mice that developed EAE in response to MOG_35–55_ showed significant increases in the secretion of IFN-gamma (*P* = 0.02) and IL-17 (*P* = 0.009) compared to resistant mice. By contrast, resistant mice were unable to mount any of these responses after the same immunization protocol. In addition, when studying serum inflammatory cytokines, we detected a significant increase of IL-17 in susceptible mice immunized with MOG_35–55_ (Figure 4(c)). This shows that downmodulation of regulatory B and T cells is related to the induction of Th1/Th17 responses in MOG-induced EAE.

## 4. Discussion

The understanding of the immune mechanisms involved in resistance/susceptibility to EAE is important, as it may contribute to identification of new therapeutic targets in MS. The role of MHC by inducing defective or appropriate presentation of the encephalitogenic peptide has been previously demonstrated [16]. However, different evidences suggest that induction of Breg may also contribute to the resistance to EAE. They are necessary to induce Treg mediated recovery from EAE [15]. Moreover, depletion of Breg exacerbates EAE symptoms and increases encephalitogenic T cell influx into the CNS [11]. In addition, induction of Breg in MOG-immunized-C57BL/6 mice treated with antibiotics ameliorates EAE course, and this effect can be transferred to other C57BL/6 mice by passive transfer of these B cells. Here, we aimed to explore if Breg may be a MHC-independent mechanism for inducting resistance to MOG-induced EAE. We explored this in C57BL/6, a congenic mouse strain susceptible to MOG_35–55_-induced EAE, and in CD1 an outbred strain resistant to MOG_35–55_-induced EAE in an MHC-independent manner. We immunized both strains of mice with the encephalitogenic peptide and observed that CD1 splenocytes were incapable of proliferating when stimulated with MOG_35–55_. We next explored immunological mechanism associated with this phenomenon and observed that susceptible C57BL/6 mice developing the disease after MOG_35–55_ immunization downregulate Breg, while CD1 resistant mice show a significant increase of these cells upon immunization with the same peptide. This strongly suggests that Breg play a role in the induction of resistance to EAE in CD1 mice.

Breg may regulate inflammatory responses by themselves and by inducing Treg [15]. It was recently shown that Treg are less suppressive in patients with MS [17]. In addition, an increase of Treg may prevent the onset of EAE [18]. In the present study we found that, in parallel with Breg findings, resistant mice upregulated Treg. This seems to indicate that Treg also have a role in resistance to MOG-induced EAE.

However, the immunization of C57BL/6 mice with the PLP_139−151_ peptide, which is incapable of inducing EAE in this strain in an MHC-dependent manner [19], did not induce significant changes in Breg or Treg subsets. We found no significant changes in both Breg and Treg. This clearly shows that when MHC molecules impede the correct peptide presentation, regulatory responses are not triggered as it happens in the outbred mice.

To further study differences between resistant and susceptible mice, we explored effector responses in the same series of mice. It has been reported that antibodies may have beneficial or detrimental effects in demyelinating diseases [20, 21]. In MOG-induced EAE model they are not related to the initiation of the disease and it remains controversial if they can influence disease severity [22, 23]. We tested the presence of anti-MOG_35–55_ IgM and IgG antibodies in serum of resistant and susceptible mice. There were no significant increases in anti-MOG_35–55_ IgG or IgM responses in MOG_35–55_-immunized mice of either susceptible or resistant strains. This shows that IgM or IgG anti-MOG_35–55_ antibodies do not play a role in inducing susceptibility to MOG_35–55_-induced EAE.

We next explored different spleen lymphocyte subsets. Previous reports showed that CD4+ T cells are required for the initial induction of progressive EAE and that the disease is downregulated by CD8+ T cells [24]. Here, we did not find differences in total CD4+ and CD8+ T cell percentages between any of the groups of resistant and susceptible mice. To further discriminate different T cell responses, we analyzed effector Th1 and Th17 subsets. It has been reported that IL-17 impairs the integrity of the blood brain barrier in EAE [25] and that a rise of spleen Th17 cells prolongs the disease [26]. Moreover, a decrease in IL-17 levels in supernatants of MOG_35–55_-activated splenocytes runs in parallel with reduced demyelination and axonal damage in mice with EAE [27]. We did not find significant differences in the percentages of CD4+ cells secreting IFN-gamma or IL-17 in nonimmunized mice. Conversely, we observed that susceptible mice that developed EAE in response to MOG_35–55_ showed significant increases in the secretion of IFN-gamma and IL-17. By contrast, resistant mice were unable to mount any of these responses after the same immunization protocol. In addition, serum levels of IL-17 were increased in susceptible mice immunized with MOG_35–55_. These data confirm the importance of the induction of Th1/Th17 responses in MOG-induced EAE.

In conclusion, our data show that upregulation of B and, to a lesser extent, of Treg, is closely associated with MHC-independent resistance to MOG-induced EAE in CD1 mice and with the abrogation of both Th1 and Th17 responses, which have a critical role in the development of the disease. These data help to ascertain the regulatory mechanisms than can downmodulate inflammatory responses in demyelinating diseases.

## Figures and Tables

**Table 1:** Titers of anti-MOG serum antibodies and T cell and B cell percentages in spleen of mice resistant (CD1) and susceptible (C57BL/6) to MOG_35–55_ induced EAE.

	Nonimmunized			MOG_35–55_-immunized			PLP_139–151_-immunized		
	C57BL/6	CD1	*P*	C57BL/6	CD1	*P*	C57BL/6	CD1	*P*
MOG-IgG (OD_492 nm_)	0.24 ± 0.02	0.17 ± 0.07	0.59	0.12 ± 0.03	0.16 ± 0.03	0.34	0.27 ± 0.07	0.07 ± 0.03	0.11
MOG-IgM (OD_492 nm_)	0.15 ± 0.03	0.06 ± 0.04	0.10	0.12 ± 0.07	0.05 ± 0.02	0.62	0.04 ± 0.01	0.06 ± 0.03	0.47
% CD4+ T cells	17.8 ± 0.96	13.5 ± 1.63	0.11	17.04 ± 3.22	12.8 ± 3.12	0.48	19.8 ± 3.33	11.5 ± 1.79	0.11
% CD8+ T cells	27.4 ± 1.53	20.3 ± 2.78	0.11	28.0 ± 3.30	24.3 ± 1.69	0.34	21.5 ± 1.02	18.0 ± 3.34	0.34

We studied nonimmunized mice and mice immunized with MOG_35–55_ and PLP_139–151_. We used eight animals per condition. Values are expressed as mean ± standard error. No differences were found in the levels of IgM or IgG anti-MOG_35–55_ antibodies expressed as optical density at 492 nm, in the percentages of CD4+ and CD8+ T lymphocytes, and total B cells (B220+) between the different experimental groups.
